# Intermediate filament-like proteins in bacteria and a cytoskeletal function in *Streptomyces*

**DOI:** 10.1111/j.1365-2958.2008.06473.x

**Published:** 2008-10-09

**Authors:** Sonchita Bagchi, Henrik Tomenius, Lyubov M Belova, Nora Ausmees

**Affiliations:** 1 Department of Cell and Molecular Biology, Uppsala UniversityBox 596, 75124 Uppsala, Sweden; 2 Department of Materials Science and Engineering, The Royal Institute of TechnologyBrinellvägen 23, 10044 Stockholm, Sweden

## Abstract

Actin and tubulin cytoskeletons are conserved and widespread in bacteria. A strikingly intermediate filament (IF)-like cytoskeleton, composed of crescentin, is also present in *Caulobacter crescentus* and determines its specific cell shape. However, the broader significance of this finding remained obscure, because crescentin appeared to be unique to *Caulobacter*. Here we demonstrate that IF-like function is probably a more widespread phenomenon in bacteria. First, we show that 21 genomes of 26 phylogenetically diverse species encoded uncharacterized proteins with a central segmented coiled coil rod domain, which we regarded as a key structural feature of IF proteins and crescentin. Experimental studies of three *in silico* predicted candidates from *Mycobacterium* and other actinomycetes revealed a common IF-like property to spontaneously assemble into filaments *in vitro.* Furthermore, the IF-like protein FilP formed cytoskeletal structures in the model actinomycete *Streptomyces coelicolor* and was needed for normal growth and morphogenesis. Atomic force microscopy of living cells revealed that the FilP cytoskeleton contributed to mechanical fitness of the hyphae, thus closely resembling the function of metazoan IF. Together, the bioinformatic and experimental data suggest that an IF-like protein architecture is a versatile design that is generally present in bacteria and utilized to perform diverse cytoskeletal tasks.

## Introduction

Research of the two past decades has revealed that bacterial cells are architecturally surprisingly complex. Bacteria, like eukaryotes, use protein filaments for spatial organization of their cells, and a large variety of cytoskeletal elements has already been identified in bacteria (Graumann, 2007). Best studied of these are the FtsZ-and MreB-family proteins, which are structurally and evolutionarily related to tubulin and actin of eukaryotic organisms. FtsZ and MreB are widespread and conserved in bacteria and fulfil important functions, including spatial orchestration of cell division, growth and morphogenesis [recently reviewed in Michie and Lowe (2006),Cabeen and Jacobs-Wagner (2007),Graumann (2007),Harold (2007),Pichoff and Lutkenhaus (2007),Thanbichler and Shapiro (2008)]. The morphogenetic function of the bacterial cytoskeleton appears to depend greatly on its ability to recruit and spatially organize proteins involved in the synthesis of the cell wall peptidoglycan (PG). PG consists of long glycan strands cross-linked by short peptide side-chains into one huge molecule encasing the cell and functions as an exoskeleton to maintain cell shape and to withstand turgor pressure. At the same time, turgor pressure may act as a driving force in cell wall expansion during growth (Koch, 1985; Harold, 2002). Thus, according to this model, growth and morphogenesis are intimately coupled in bacteria. By allowing cell wall expansion only at certain positions dictated by cytoskeletal structures and driven by turgor pressure (and\or other forces), different shapes can be generated (Cabeen and Jacobs-Wagner, 2007; Harold, 2007). The helical cytoskeletal structure formed *in vivo* by the bacterial actin MreB defines a cylinder and thus determines the common rod shape of many bacteria (Jones *et al*., 2001; Kruse *et al*., 2003; Figge *et al*., 2004; Dye *et al*., 2005; Carballido-Lopez, 2006; Carballido-Lopez *et al*., 2006; Divakaruni *et al*., 2007; Mohammadi *et al*., 2007). Ends of the cell cylinder are capped by hemispherical poles, generated by a cell division process guided by the action of bacterial tubulin FtsZ, which forms a constricting ring structure at the cell division site (Bi and Lutkenhaus, 1991; Daniel and Errington, 2003; Varma and Young, 2004; Margolin, 2005; Aaron *et al*., 2007). Cocci generally do not contain MreB and are made up of two poles created by FtsZ-directed cell division (Jones *et al*., 2001; Pinho and Errington, 2003). However, the cell shape arsenal of bacteria contains much more than just spheres and straight rods. Obviously, other spatial organizers besides MreB and FtsZ must exist in bacteria to determine more complicated forms. A step further in understanding bacterial morphogenesis was the identification of the intermediate filament (IF)-like protein crescentin in an aquatic bacterium with a characteristic crescent-like cell shape, *Caulobacter crescentus* (Ausmees *et al*., 2003). The laterally localized crescentin cytoskeleton converts the FtsZ-and MreB-dependent simple straight rod into a curved or helical rod, depending on the length of the cell (Ausmees *et al*., 2003). Surprisingly, despite the remarkable architectural and biochemical relatedness of crescentin to IF proteins, a sequence similarity search failed to reveal crescentin homologues in other bacteria. Thus, the additional morphogenetic factors in other MreB\FtsZ-bacteria with complex shapes remain to be uncovered. How general is the above outlined morphogenetic model based on the actions of MreB, FtsZ and additional cytoskeletal elements, such as crescentin? Remarkably, at least one large and important group of bacteria, the actinomycetes, seem to use a different *modus operandi* for cell growth and morphogenesis that is independent of MreB and FtsZ. These bacteria grow in a polarized fashion: rod-shaped species, such as *Mycobacterium* and *Corynebacterium* assemble new cell wall at both cell poles, while filamentous ones, such as *Streptomyces*, grow by hyphal tip extension analogously to how filamentous fungi grow (Daniel and Errington, 2003; Flärdh, 2003a,Flb; Ramos *et al*., 2003; Nguyen *et al*., 2007; Letek *et al*., 2008). The actinomycetes exhibit extraordinary diversity, regarding their morphology (from simple coccoid to complex branching and multicellular filaments), lifestyle (e.g. pathogenic like *Mycobacterium* or symbiotic like *Frankia*), physiology, metabolism (e.g. production of a multitude of secondary metabolites by *Streptomyces*) and colonization of various environmental niches (Ventura *et al*., 2007). The coiled coil protein DivIVA seems to have an important role in generation of polarity in these bacteria (Flärdh, 2003a; Letek *et al*., 2008), but other than that, the molecular mechanisms underlying the diverse morphologies of actinomycetes are relatively poorly understood.

One of the main objectives of the present study was to investigate the incidence of the IF-like function in bacteria, prompted by the intriguing observation that crescentin, the bacterial counterpart of metazoan IF, did not appear to be conserved in bacteria. Was acquisition of crescentin by *C. crescentus* just an isolated event or might a similar function, perhaps carried by structurally similar proteins albeit with diverging primary sequences, be more common? Here we demonstrate that proteins with a basically IF-or crescentin-like architecture are in fact present and widespread in bacteria. Experimental analysis of a conserved group of such proteins from actinobacteria demonstrated that IF-like biochemical properties accompanied the basic IF-like architecture of these proteins and also revealed a novel cytoskeletal function in actinobacterial growth and morphogenesis. Together our data suggest that an IF-like cytoskeleton, based on a versatile structural element of a segmented coiled coil rod, is more widespread in bacteria than previously thought.

## Results

### Proteins with a potential segmented coiled coil rod domain are widespread in bacteria

**Fig. 1:**
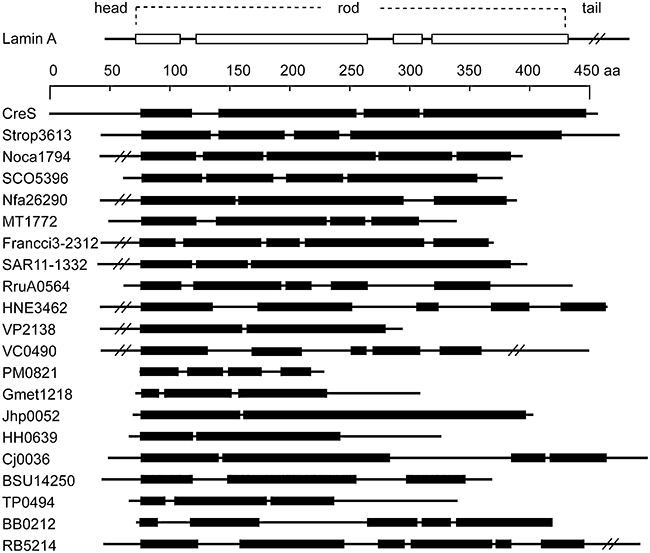
Architectures of bacterial rod-domain proteins. The tripartite building plan of a human IF protein nuclear lamin A is depicted at the top of the figure. The scale bar refers to amino acid residues of crescentin (CreS), the IF-like protein from *C. crescentus.* Other proteins are drawn in scale, and aligned in respect to their first coiled coil domains. Boxes represent domains in coiled coil conformation and lines non-coiled coil sequences. Long head or tail domains are in some occasions truncated. Designations refer to locus tags of respective proteins from the following species (in order of occurrence): *S. tropica, Nocardioides* sp. JS614, *S. coelicolor, N. farcinica, M. tuberculosis, Frankia* sp. Ccl3, *P. ubique*, *R. rubrum, H. neptunium, V. parahaemolyticus, V. cholerae, P. multocida, G. metallireducens, H. pylori, H. hepaticus, C. jejuni, B. subtilis, T. pallidum, B. burgdorferi, R. baltica*.

**Fig. 2:**
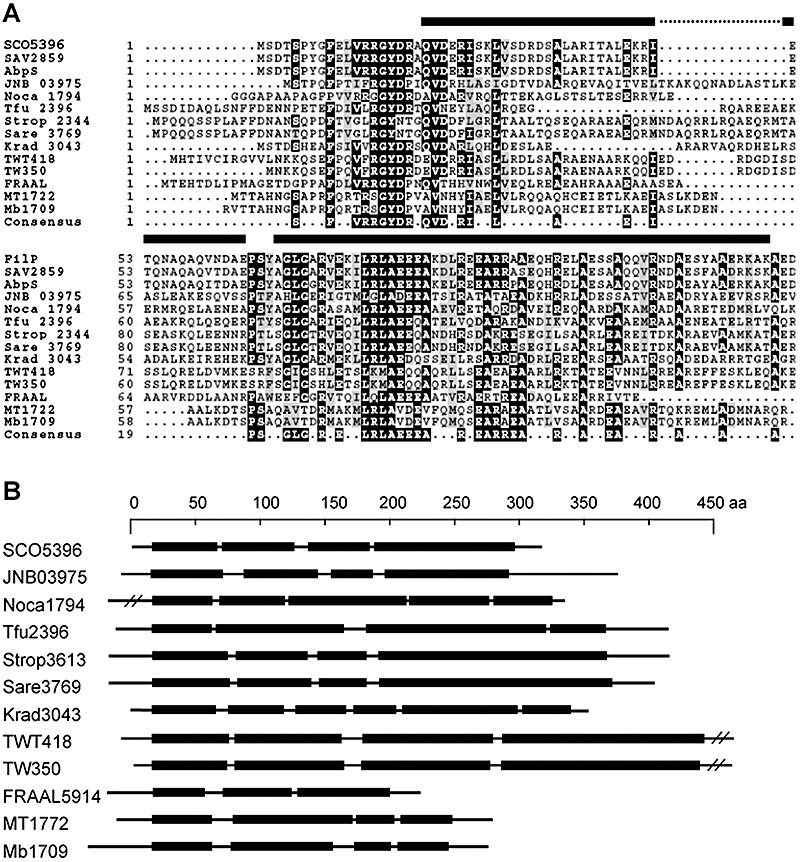
Conserved sequence motifs define a protein family in actinomycetes. A. MULTALIGN alignment (Corpet, 1988) of the N-termini of the FilP-family proteins. AbpS designates the Avicel-binding proteins from *S. reticuli*. Other proteins are identified by their locus tags shown to the left of the alignment. SCO –*S. coelicolor*, SAV –*S. avermitilis*, Krad –*Kineococcus radiotolerans*, Noca –*Nocardioides* sp., Tfu –*Thermobifida fusca*, FRAAL –*Frankia alni*, JNB –*Janibacter* sp., TWT – T*ropheryma whipplei* strain Twist, TW –*Tropheryma whipplei* strain TW, MT –*M. tuberculosis*, Mb –*M. bovis*, Strop –*S. tropica*, Sare –*Salinispora arenicola*. Black bars represent the positions of the two first coiled coil domains of SCO5396 (FilP). Multiple alignment was performed with default settings and the output order reflects the relatedness of the input sequences. B. Schematic representation of the various coiled coil architectures of the proteins belonging to the conserved family in (A). Black bars represent coiled coil domains, lines represent non-coiled coil sequences. Long head and tail domains are truncated in some cases.

Despite poor sequence conservation all eukaryotic IF proteins share a similar building plan, consisting of a central rod domain of alternating coiled coil segments and linkers, flanked by more globular head and tail domains (Fig. 1, top) (Herrmann and Aebi, 2004). The rod domain is absolutely essential for filament formation (Parry *et al*., 2007). The bacterial IF-like protein crescentin also possesses an apparent rod domain with a different arrangement of coiled coil segments and linkers (Fig. 1) yet exhibits remarkably IF-like biochemical properties and has a cytoskeletal function (Ausmees *et al*., 2003). We chose arbitrarily 26 bacterial genomes to survey for the presence of rod-domain proteins among the encoded proteomes (Table 1). A rod domain was defined as a sequence of more than 80 amino acids in coiled coil conformation, positioned either as one continuous block or interrupted by short non-coiled coil sequences. An additional condition was that the candidates should contain no known functional domains, such as a signal sequence, transmembrane segments, enzymatic activity, etc. Out of 26 genomes 21 encoded at least one candidate protein with above mentioned properties, and 16 genomes encoded several (Table 1). One putative crescentin-like rod-domain protein from each genome is schematically shown in Fig. 1. The analysed genomes represent distant phylogenetic groups, suggesting that proteins with a rod-like architecture are widespread among bacteria. Further database searches revealed a conserved rod-domain protein family with members from at least 14 actinomycete species. The hallmark of this family was a pair of clearly conserved sequence motifs at the N-terminal borders of the two first coiled coil segments, regardless of the different lengths of the respective segments and intervening linkers in individual proteins (Fig. 2). These conserved motifs are not discernible in crescentin. However, the basically crescentin-like architecture of the actinomycete proteins and their lack of other known domains suggested that they might similarly have a structural or cytoskeletal function *in vivo*, so we subjected this family of proteins to further analysis.

**Table 1:** Summary of COILS prediction of 26 bacterial genomes.

Species	Class	> 100^a^	> 200^a^	Rod^b^
*S. coelicolor*	Actinobacteria	15	6	3
*S. tropica*	Actinobacteria	11	4	4
*N. farcinica*	Actinobacteria	10	5	3
*Frankia sp. CcI3*	Actinobacteria	5	0	2
*Nocardioides sp. JS614*	Actinobacteria	4	2	2
*M. tuberculosis*	Actinobacteria	2	1	2
*R. rubrum*	Alphaproteobacteria	5	1	2
*H. neptunium*	Alphaproteobacteria	5	1	2
*C. crescentus*	Alphaproteobacteria	4	2	1
*Maricaulis maris*	Alphaproteobacteria	3	1	0
*Agrobacterium tumefaciens*	Alphaproteobacteria	3	1	0
*P. ubique*	Alphaproteobacteria	3	2	2
*G. metallireducens*	Deltaproteobacteria	12	2	1
*Desulfovibrio desulphuricans*	Deltaproteobacteria	7	0	0
*H. pylori*	Epsilonproteobacteria	18	3	4
*C. jejuni*	Epsilonproteobacteria	5	0	5
*H. hepaticus*	Epsilonproteobacteria	1	0	2
*V. parahaemolyticus*	Gammaproteobacteria	11	1	2
*P. multocida*	Gammaproteobacteria	10	1	2
*E. coli 536*	Gammaproteobacteria	8	2	0
*V. cholerae*	Gammaproteobacteria	10	2	1
*B. subtilis str. 168*	Bacillales	13	5	1
*R. baltica*	Planctomycetacia	22	7	2
*L. interrogans*	Spirochaetes	9	2	0
*B. burgdorferi*	Spirochaetes	7	1	2
*T. pallidum*	Spirochaetes	5	2	1

a Number of proteins with more than 100 and 200 amino acid residues in coiled coil conformation encoded in each genome.

b Number of proteins with a putative rod domain and no known function encoded in the respective genome.

### Rod-domain proteins from actinomycetes form filaments in vitro

**Fig. 3:**
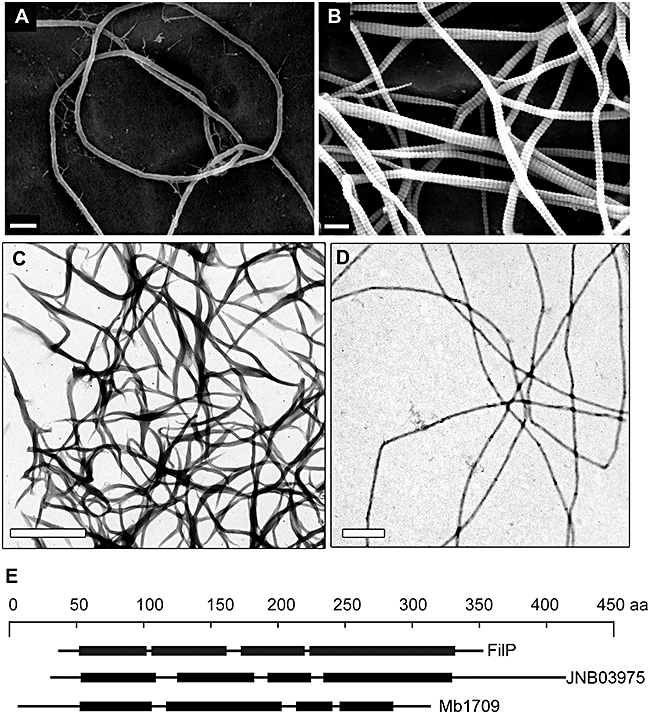
*In vitro* filaments formed by rod-domain proteins of actinomycetes. A and B. Scanning electron micrographs of filaments formed by *S. coelicolor* protein SCO5396 (FilP) in 50 mM TrisHCl at pH 7.0. A smooth non-branching filament is shown in A and the striated branching filaments are shown in B. C and D. Transmission electron micrographs of negatively stained filaments formed by *Janibacter* sp. protein JNB03975 and by *M. bovis* protein Mb 1709 respectively. E. Schematic representation of the various rod-domain structures of the proteins in A-C. Size bars represent 200 nm for A and B, and 1 μm for C and D.

A characteristic property of cytoskeletal proteins in general is to form filamentous structures *in vitro*. Differently from actin and tubulin, IF proteins and crescentin do not require binding of nucleotide or other cofactors but polymerize spontaneously into regular filaments *in vitro* upon denaturation and subsequent renaturation in physiological buffers (Steinert *et al*., 1976; Domingo *et al*., 1992; Ausmees *et al*., 2003; Parry *et al*., 2007). Because members of the actinobacterial rod-domain family displayed considerably different arrangements of coiled coil segments and linkers despite conserved sequence motifs (Figs 2B and 3E), we chose three proteins with distinct domain architectures for biochemical analysis. Proteins corresponding to locus tags SCO5396 from *Streptomyces coelicolor* A3(2), Mb 1709 from *Mycobacterium bovis* and JNB03975 from *Janibacter* sp. were purified as recombinant N-terminally polyhistidine-tagged proteins in denatured form and subjected to renaturation conditions. Scanning and transmission electron microscopy revealed that, indeed, all three proteins assembled into filaments, albeit with different morphologies, in buffers with neutral pH and physiological salt concentrations (Fig. 3). It is also known that filament morphology can vary depending on the individual IF protein and buffer conditions (Goulielmos *et al*., 1996; Geisler *et al*., 1998; Ausmees *et al*., 2003). The 35 kDa rod-domain protein from *S. coelicolor* produced two distinct types of filaments in a 50 mM TrisHCl buffer at pH 7.0. A long smooth and rope-like filament with a diameter of ˜60 nm (Fig. 3A) constituted the less frequent type. The even width and lack of branches indicate that this is a stable polymer, unable to add subunits\filaments laterally. The more abundant type consisted of branching striated filaments of varying diameter (Fig. 3B). The latter filaments visually resembled those formed by nuclear lamins under similar conditions (Stuurman *et al*., 1998). The relative abundance of smooth and striated filaments did not change significantly in buffers containing 150 mM NaCl or 20 mM MgCl_2_. The Mb 1709 protein from *M. bovis* formed a network of laterally aggregating smooth filaments (Fig. 3C), whereas JNB03975 from *Janibacter* sp. formed smooth filaments with a constant diameter of approximately 40 nm, which did not aggregate laterally (Fig. 3D). Formation of *in vitro* filaments by three members with distinctly different architectures of the rod domains and belonging to phylogenetically divergent species of *Actinobacteria* suggests that assembly into regular structures *in vitro* is a common property of this conserved protein family (Figs 2 and 3). Because *S. coelicolor* is a genetically tractable model organism for actinomycetes, we chose SCO5396 (hereafter called *filP* for *fil*ament-forming *p*rotein) for further *in vivo* studies.

### FilP forms filamentous structures *in vivo*

**Fig. 4:**
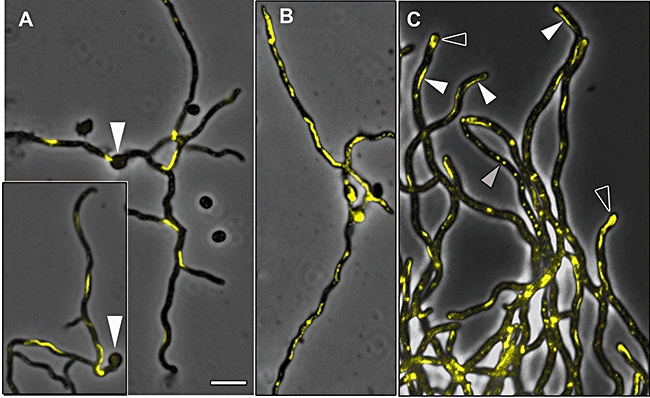
FilP-EGFP forms filamentous structures in growing *S. coelicolor* hyphae. A–C. Overlays of fluorescence and phase contrast micrographs, FilP-EGFP fluorescence is false coloured yellow. Young vegetative hyphae (12 h) of the *filP-egfp* strain grown in solid MS agar medium show pronounced filamentous structures of FilP-EGFP (A). White arrowheads mark germinated spores. Young vegetative hyphae (12 h) of the merodiploid *filP*^+^*\filP-egfp* strain are shown in B. The hybrid FilP\FilP-EGFP filaments are longer and display weaker fluorescence than those of FilP-EGFP. Hyphae of the merodiploid *filP*^+^*\filP-egfp* strain grown in liquid YEME medium for 14 h are slightly larger than those grown on solid MS medium and contain several types of fluorescent structures (C). White arrowheads indicate FilP\FilP-EGFP filaments, open arrowheads indicate diffuse apical spots, and grey arrowheads indicate condensed foci. Size bar corresponds to 4 μm in all panels.

*Streptomyces coelicolor* is a soil-dwelling filamentous and truly multicellular bacterium with a complex lifestyle [reviewed in Chater and Losick (1997),Chater (2000),Flärdh (2003a),Elliot *et al*. (2007)]. First, a network of apically growing and branching vegetative hyphae invade the growth substrate. In response to exhaustion of the nutrients and after complex signalling, submerged vegetative hyphae give rise to aerial hyphae which break the water–air interface. Aerial hyphae then develop into spore chains and ultimately produce unicellular heat-and desiccation-resistant spores. We fused the enhanced green fluorescent protein (EGFP) to the C-terminus of FilP for its visualization *in vivo*. Samples for live-cell fluorescence microscopy were withdrawn from developing cultures containing *filP-egfp* grown for 12, 24, 36 and 48 h to visualize all different cell types: germinating spores, vegetative mycelium, aerial hyphae and spore chains. A *filP* deletion strain in which *filP-egfp* was expressed from the native promoter at an ectopic locus in the chromosome (NA446 in Table S1) exhibited prominent fluorescent filamentous structures already in vegetatively growing hyphae (Fig. 4A). We also detected FilP-EGFP filaments in immature and still growing aerial hyphae, but not in mature spore chains in which growth had stopped (data not shown), suggesting that FilP might be involved in normal hyphal growth or germination. However, the *filP-egfp* strain had a slight morphological defect similar to that of the †*filP* mutant (see below), suggesting that the FilP-EGFP filaments were not fully functional. A merodiploid *filP*^+^*\filP-egfp* strain (NA282 in Table S1), on the other hand, was morphologically indistinguishable from the wild-type and displayed different spatial organization of FilP-EGFP fluorescence compared with the *filP-egfp* strain. Redistribution of the FilP-EGFP fluorescence in the presence of the wild-type FilP protein indicates that the tagged and untagged FilP proteins form hybrid structures. Figure 4C shows young vegetative hyphae of the merodiploid strain grown in liquid culture and containing various FilP\FilP-EGFP structures. First, the tip regions of nearly all young hyphae exhibited strong FilP-EGFP fluorescence as a distinct filament and\or as a more diffuse spot (arrowheads in Fig. 4C). This consistent localization to apical areas was not observed in the *filP-egfp* strain, which produces no wild-type FilP (Fig. 4A). FilP\FilP-EGFP filaments were also present in tip-distal regions. Similar localization was observed in hyphae grown on solid medium (Fig. 4B). Hybrid FilP\FilP-EGFP filaments were often longer (compare Fig. 4A with B) and displayed weaker fluorescence intensity than those of FilP-EGFP. Second, condensed spots or foci were also seen in most hyphae (Fig. 3C). Unfortunately, we were unable to localize the native FilP in the wild-type situation. An attempt to produce a polyclonal anti-FilP antiserum was unsuccessful, and the fusion of a short antigenic FLAG tag to the C-terminus of FilP rendered it partially non-functional. However, the wild-type morphology and growth rate of the merodiploid *filP*^+^*\filP-egfp* strain suggest that the hybrid FilP\FilP-EGFP structures are functional. This closely resembles the *in vivo* behaviour of tagged and untagged crescentin derivatives. Filaments formed entirely by crescentin-EGFP are short, brightly fluorescent and non-functional. Addition of the wild-type crescentin causes redistribution of crescentin-EGFP into dimmer and slightly discontinuous hybrid crescentin\crescentin-EGFP structures, which stretch from pole to pole along the concave side of the cells and are able to cause cell curvature (Ausmees *et al*., 2003). Thus, the resemblances in the domain architecture and *in vitro* properties of FilP and crescentin extend also to their behaviour *in vivo*.

We also considered the possibility that FilP might form hybrid structures with other rod-domain proteins SCO3114 and SCO5397 of *S. coelicolor in vivo* (Table 1). SCO3114 displayed a weak sequence similarity to FilP but lacked the characteristic N-terminal sequence motifs. SCO5397 (a very large coiled coil protein of more than 1300 aa) was not conserved in other bacteria. *SCO5397-egfp* strain and a merodiploid *SCO3114*^+^*\SCO3114-mcherry* strain (NA360 and NA399 respectively, in Table S1) exhibited wild-type morphology and displayed fluorescence patterns very different from those of the *filP-egfp* strains. SCO5397-EGFP localized in a weak, indistinct punctate pattern, and SCO3114-mCherry (mCherry is a red fluorescent protein) displayed a relatively strong diffuse signal (Fig. S1). As FilP did not colocalize with other rod-domain proteins of *S. coelicolor* we assume that a straightforward functional redundancy of these proteins is unlikely, although we cannot exclude some overlap in their respective cellular roles.

### *filP* deletion causes defects in growth and morphology

**Fig. 5:**
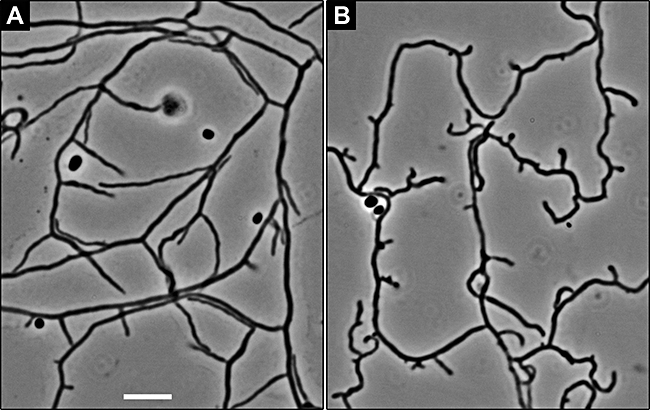
Deletion of *filP* causes a morphological defect. A and B. Live hyphae of the wild-type strain M145 (A) and the †*filP* mutant strain (B) grown for 20 h in the angle between an inserted coverslip and the MS agar surface and visualized by phase contrast microscopy.

To address the biological function of the FilP filamentous structures, we studied a strain in which *filP* was replaced by an apramycin resistance cassette (†*filP*, NA335 in Table S1). First, the †*filP* strain had a pronounced growth defect. For example, growth tests in various liquid and solid media showed that after inoculation of an equal number of viable spores, the mutant accumulated only 49–78% of the biomass of the wild-type strain in 30–36 h (data not shown). Total growth is affected by germination efficiency of the spores, the linear growth rate and the branching frequency of the hyphae. Growth curves (Fig. S2) indicated that biomass accumulation started with a delay and proceeded more slowly in the †*filP* strain compared with wild-type in a liquid medium. As we did not observe overt differences in the frequency of branching between the †*filP* and the wild-type strains, we assumed that a combination of delayed germination and slower linear growth was responsible for the total growth defect of the †*filP* strain. Slower growth of the mutant on solid media was also evident, although normal amounts of dark grey spores were finally produced (approximately a day later than the wild-type), indicating that sporulation was not significantly affected. Furthermore, vegetative hyphae of the mutant showed a characteristic distorted morphology when grown in the angle between an inserted coverslip and the surface of the solid agar medium for microscopic observation (Fig. 5B). Wild-type hyphae grew in a straight fashion under the same conditions (Fig. 5A, see also Fig. S3). A similar difference in morphology between the strains was observed also in liquid cultures (data not shown), thus ruling out the possibility that the apparent morphological differences were caused by different surface adhesion properties of the two strains. Aerial hyphae of the †*filP* strain, however, did not display any striking morphological defects. Both the morphological and growth defects of the †*filP* strain were complemented by addition of wild-type *filP in trans*, confirming that the mutant phenotypes were caused by the lack of *filP* (data not shown).

### Atomic force microscopy shows that *filP* is needed for normal rigidity of the hyphae

**Fig. 6:**
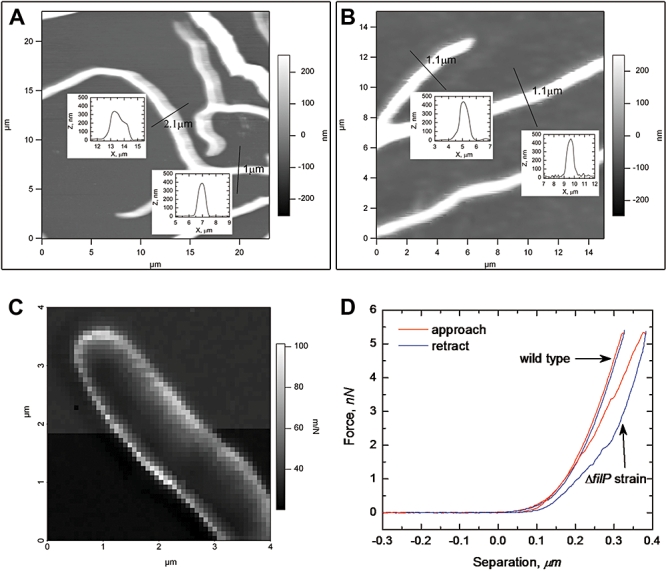
Deletion of *filP* causes changes in the visco-elastic properties of *S. coelicolor* cells. A and B. Topological images of live hyphae of the wild-type M145 (B) and the †*filP* strain (A) grown as in Fig. 4. Insets show height profiles of the hyphae at positions marked with perpendicular lines, indicating that mutant hyphae undergo deformation in the imaging process, whereas wild-type hyphae maintain their form. C. An image of a tip of a wild-type hypha reconstructed using data from 80 × 80 force curves. Each pixel corresponds to a compliance value calculated from an individual force curve and depicted in greyscale, as shown by a scale bar to the right. Thus, areas of the sample characterized by dark pixels are relatively stiff (low compliance), and areas with lighter pixels are softer (high compliance). D. Single representative force curves of wild-type and *†filP* hyphae showing that wild-type hyphae are less compliant than those of the mutant. The hysteresis effect (manifested as a loop between the load and unload curves) was consistently present in all force curves of the mutant hyphae, and absent in those of the wild-type.

The PG sacculus of bacteria is the most important cellular component to maintain cell shape and integrity, and many mutations affecting assembly of PG are known to cause altered cell morphology. Thus, we speculated that the morphological defect of the †*filP* hyphae is perhaps caused by a weakened PG exoskeleton, which renders the hyphae less rigid and is unable to elongate in a straight fashion typical to those of the wild-type. To test this, we probed living vegetative hyphae of the wild-type and the †*filP* strains by atomic force microscopy (AFM). First, topological images were collected of both strains in a completely hydrated state by using the contact imaging mode (Fig. 6A and B and Fig. S3). Interestingly, †*filP* hyphae seemed to yield more under the tip pressure and shear forces, as compared with wild-type hyphae. Height profiles in Fig. 6A show significant distortion of the †*filP* hyphae, especially in the parts lying perpendicular to the fast scan direction (see Supporting information text for additional information). In contrast, the wild-type hyphae remained undistorted and exhibited uniform height and width (Fig. 6B). This indicates that †*filP* hyphae are more deformable and ‘softer’ than those of the wild-type. Further, to measure the elastic properties of the hyphae, we recorded force curves as the tip of the cantilever approached, indented the hyphal surface for about 100 nm and then retracted. Force volume (an array of force curves) was recorded over several areas containing apical parts of wild-type or †*filP* hyphae. Figure 6C displays an image reconstructed using compliance values calculated from an 80 × 80 array of force curves over a tip of a wild-type hypha. Compliance is the inverse of stiffness and the latter is defined as *dFdI*^-1^ with *F* being the force and *I* being the indentation. Because of the pyramidal shape of the tip the edges of a cell experience deformation simultaneously in downwards and sidewise direction, which results in high apparent compliance. To avoid edge effects 50 force curves along a line running parallel to the long axis of the hyphae in the highest part of the hyphal cylinder were used for analysis, resulting in average compliance values of 34.4 m N^-1^ and 44.1 m N^-1^ for wild-type and †*filP* hyphae respectively. Single typical force-separation curves of †*filP*, and wild-type hyphae are shown in Fig. 6D. The steeper slope of the wild-type force curve implies lower compliance of the wild-type hypha compared with the †*filP* hypha. As both hyphae were indented by the same cantilever, this indicates that the hyphae of the wild-type strain are stiffer than those of the †*filP* strain. Another significant observation is that the ‘load’ and ‘unload’ curves match reproducibly over the entire surface of the wild-type hyphae (Fig. 6D). This indicates that the wild-type hyphae undergo a fully reversible elastic deformation and revert to their original shape as the tip retracts. On the other hand, the †*filP* hyphae seemed to suffer a more persistent deformation, indicated by the significant hysteresis effect consistently present in all force curves (manifested as a loop between the load and unload curves as the cells demonstrate a delayed elastic recovery after deformation, Fig. 6D). This is in agreement with other AFM data and can be explained by a softer cell surface and\or by lower turgor pressure of the †*filP* hyphae. Thus, our data indicate that the FilP cytoskeleton affects the mechanical properties of the cells. This is consistent with the current model of bacterial morphogenesis, according to which cytoskeletal elements control mechanical properties of the cell by spatially orchestrating the deposition of the stress-bearing exoskeleton. Nevertheless, this to our knowledge the first time the impact of bacterial cytoskeleton has been demonstrated by direct probing of the physical properties of the cells.

## Discussion

### Diversity of putative IF-like proteins and cytoskeletal functions in bacteria

One of the objectives of this study was to address whether the IF-like cytoskeleton is confined to only one bacterial species or if it might be a more widespread phenomenon. The latter notion was evoked by the high degree of diversity displayed by the sequences of IF proteins. For example, there is only approximately 30% sequence identity between the rod domains of human and fruit fly lamins, and 27% between two human IF proteins lamin A and cytokeratin 18. Also, individual IF perform quite different functions and are present in different cell types. This is in strong contrast with the strict conservation of the sequences and functions of the actin and tubulin proteins. Apparently, the basic structure of IF proteins, consisting of a segmented coiled coil rod (supports assembly into cytoskeletal filaments) flanked by the head and tail domains (accommodate additional functional motifs), is a versatile design and can be adapted to various cellular functions. This could be a reason why the evolution of IF is less constrained than that of actin and tubulin. Thus, to address our objective, we first estimated the occurrence of the basic IF architecture among bacterial proteins. Our analysis suggests that proteins with a segmented coiled coil rod domain are present in many diverse species (Figs 1 and 2). However, a cytoskeletal function has to be shown experimentally in each case. We chose a protein (FilP) from a conserved actinobacterial rod-domain family and demonstrated that it indeed performed a cytoskeletal function in *S. coelicolor*. Interestingly, members of that sequence-wise conserved actinomycete protein family display different architectures of the segmented rod domain (Fig. 2). Nevertheless, three tested members, representing different architectures, were able to form regular filaments *in vitro*. This supports the idea of a versatile coiled coil rod, which does not have to conform to a strictly defined sequence and architecture to support filament formation and a cytoskeletal function. Rather, the specific architecture is probably tailored for the specific function of each protein. The common occurrence of rod domains in bacteria together with our experimental data suggest that diverse bacteria may possess a filament system composed of proteins sharing the basic design of IF elements. However, it is not clear whether these proteins originate from a common ancestor or have been evolved independently.

Our analysis was restricted to previously uncharacterized rod-domain proteins without known functional domains in order to identify crescentin-like cytoskeletal elements. However, there are also examples of proteins in which rod domains are coupled to a known function. For example, rod-domain proteins TlpA from a *Salmonella enterica* virulence plasmid (Hurme *et al*., 1994) and Scc from *Leptospira interrogans* (Mazouni *et al*., 2006) were both shown to form filaments *in vitro* and *in vivo* in *Escherichia coli* cells. A common feature of these otherwise very different proteins is that both also bind DNA. Another example is the AglZ protein from *Myxococcus xanthus* which carries a filament-forming coiled coil module coupled to a response regulator receiver domain (Yang *et al*., 2004). Very recently, a short (only 81 aa long) coiled coil protein ZapB was also shown to form striated filaments *in vitro* and have a role in FtsZ-ring assembly and cell division in *E. coli* (Ebersbach *et al*., 2008). Thus, filament formation via coiled coil rod domains might be relevant in various functional contexts in a bacterial cell.

### FilP cytoskeleton in cell growth and morphology

The second goal was to characterize the novel cytoskeletal function identified in this study, performed by an *S. coelicolor* rod-domain protein FilP. We have shown that a *filP* mutant was affected in growth and morphology, indicating that FilP has a role in both processes. The observation that *filP* mutant hyphae were more compliant under direct mechanical probing with AFM suggests that the softness of the cells might lie behind both defects – the distorted morphology and the slower growth. One can envision that mechanical forces of the environment, such as turbulence in liquid or roughness of the solid substrate, would distort the softer †*filP* hyphae to a greater extent than the more rigid wild-type hyphae. Thus, frequent change of growth direction might cause the undulating morphology of the mutant hyphae, while the wild-type hyphae are able to grow in a straight fashion in similar conditions (Fig. 5). It has previously been shown that externally applied mechanical constraints induce long-persisting changes in bacterial cell shape. For example, *E. coli* cells forced to grow as cell filaments in circular microchambers retained a bent cell shape upon release into a liquid medium even after growth and cell division (Takeuchi *et al*., 2005). Reduced rigidity might also cause less efficient tip elongation of the †*filP* strain and explain its slower accumulation of biomass.

Rigidity of the bacterial cells is mainly determined by the cell wall and by turgor pressure. At present we cannot convincingly determine whether the increased compliance of the †*filP* strain is due to changes in the cell envelope structure or lower turgor pressure. As discussed above, the strategic site of new cell wall synthesis in *S. coelicolor* is the tip of a growing or emerging hypha, where also a key protein in this process, DivIVA, localizes (Flärdh, 2003a,b). Interestingly, the tip regions of growing hyphae of the merodiploid *filP*^+^*\filP-egfp* strain consistently contained a strong fluorescence signal of FilP-EGFP evident as a filamentous structure, an apical spot or both (Fig. 4B and C). Together, the presence of FilP within the zone of active growth and the abnormal softness of the cells in the absence of FilP suggest that it might have a role, although nonessential, in cell wall synthesis or maturation. Another possibility is that FilP somehow changes the composition or permeability of the cell membrane and by that affects turgor pressure and thus the rigidity. Furthermore, it cannot be excluded that FilP structures might act as a mechanical support in resemblance with the IF network in mammalian cells. Cell wall expansion in bacteria involves lytic activity in order to break already existing cross-links and incorporate new PG precursors. Thus, in the growing region additional mechanical support might be needed to compensate for the relative weakness of the exoskeleton. A similar function has very recently been proposed for a cellulose synthase-like protein CslA of *S. coelicolor* (Xu *et al*., 2008). CslA localized to the hyphal tips and was responsible for the accumulation of a β-glycan polysaccharide. The authors proposed that this polysaccharide, most likely cellulose, acts as a bandage to reinforce the growing tips of the hyphae. Interestingly, a FilP orthologue from *S. reticuli* has previously been shown to bind Avicel, a derivative of cellulose and was therefore named AbpS (Avicel-binding protein) (Walter *et al*., 1998). The authors also showed that AbpS oligomerizes (Walter and Schrempf, 2003), which agrees with our data showing that FilP forms filaments *in vitro* and *in vivo*. We assume that FilP and other proteins of the family might share the cellulose-binding ability of AbpS. As FilP is also present at the hyphal tips and in the subapical regions, it must at least partially colocalize with CslA. Future studies might clarify the significance of the cellulose-binding ability of FilP and the interaction network between the proteins populating the tip regions of elongating hyphae.

The apparent involvement of FilP in determining the mechanical properties of *S. coelicolor* hyphae relates to the functions of metazoan IF. Although IF have become more and more implicated in signalling, protein targeting and other dynamic cellular processes, one important aspect of their functions is mechanical, providing structural support to the cells and dealing with mechanical stress (Chou *et al*., 2007; Kim and Coulombe, 2007; Pekny and Lane, 2007). Indeed, several disease-causing mutations in genes encoding IF-proteins have been shown to, directly or indirectly, lead to increased fragility of affected cells and tissues (Omary *et al*., 2004; Herrmann *et al*., 2007). Similarly, the compromised rigidity of the †*filP* strain might render it more susceptible to the mechanical stresses inflicted by the environment and affect survival and fitness in nature. The presence of FilP-like proteins in other actinomycetes and *in vitro* filament formation by FilP-family proteins from *Janibacter* sp. and *M. bovis* suggest that a similar IF-like cytoskeletal function is conserved. *Janibacter* and *Mycobacterium* species display slightly irregular rod shape and are also known to adopt different morphologies under certain conditions (Martin *et al*., 1997; Ojha *et al*., 2000). It would be interesting to study the cellular roles of FilP-family proteins in non-filamentous bacteria. Further research is also needed to understand the details of FilP localization, the relevance of its different cellular structures and whether FilP shares the property of other cytoskeletal elements, such as MreB and FtsZ, to act as a spatial organizer of cell wall construction. To this end, we believe that our novel application of AFM offers a useful tool to address the roles of various cytoskeletal elements in the important task of building up a bacterial cell.

## Experimental procedures

### Bacterial strains and media

The *S. coelicolor* A3(2) and *E. coli* strains used in this work are listed in Table S1. Cultivation of *E. coli* strains was performed as described in (Sambrook *et al*., 1989). *S. coelicolor* strains were grown on mannitol soy flour agar plates (MS agar), in yeast extract-malt extract medium (YEME), in tryptone soy broth or on R2YE agar (Kieser *et al*., 2000).

### Construction of plasmids and recombinant *S. coelicolor* strains

The plasmids used are listed in Table S1. DNA manipulation and cloning were carried out according to standard protocols (Sambrook *et al*., 1989). DNA fragments to be cloned into plasmid vectors were created by PCR, and final constructs were verified by DNA sequencing. Sequences of primers used for cloning and construction of mutants and exact cloning strategies are available upon request. The PCR-targeting procedure was used for generation of *filP* gene knock-out mutant (NA335) essentially as described in Gust *et al*. (2003) (see also Table S1). To generate the merodiploid *filP*^+^*\filP-egfp* strain (NA282), the entire *filP* gene with 526 bp upstream region was first fused in frame to *egfp* resulting in plasmid pNA859. *S. coelicolor* protoplasts were then transformed with pNA859, followed by selection of transformants where homologous recombination via a single crossing-over event had created a full-length recombinant fusion allele in the wild-type locus under control of the native promoter, as well as a second full-length copy of the gene. A similar strategy was used to create the merodiploid *SCO3114*^+^*\SCO3114-mcherry* strain NA399. A derivative of the cloning vector pBluescript, containing an apramycin resistance cassette and full-length *SCO3114* fused to *mcherry* (Shaner *et al*., 2004), was used for transformation of *S. coelicolor* protoplasts. *SCO5397-egfp* strain (NA360) was constructed by insertion of pEGFP-N2 encoding a C-terminal part of SCO5397 fused to EGFP into the chromosome of M145 by homologous recombination. For generation of the *filP-egfp* strain (NA446) the insert from pNA859 encoding FilP-EGFP was subcloned into the vector pIJ82 and by conjugation introduced into strain NA335 (†*filP*) where it integrated in the chromosome at the phage ΦC31 attachment site. Mutant strains were verified by diagnostic PCR.

### Database searches

Genome sequences of *C. crescentus* CB15 (AE005673.1), *Salinispora tropica* CNB-440 (NC009380), *Nocardioides* sp. JS614 (NC008699), *S. coelicolor* A3(2) (NC003888)*, Nocardia farcinica* IFM 10152 (NC006361)*, Mycobacterium tuberculosis* CDC1551 (NC002755)*, Frankia* sp. Ccl3 (NC007777), *Pelagibacter ubique* HTCC1062 (NC007205), *Rhodospirillum rubrum* (NZAAAG00000000), *Hyphomonas neptunium* ATCC 15444 (NC008358), *Vibrio parahaemolyticus* RIMD 2210633 (NC004603), *Vibrio cholerae* (NC002505, NC002506)*, Pasteurella multocida* (NC002663)*, Geobacter metallireducens* (NZAAAS00000000), *Helicobacter pylori* J99 (NC000921)*, Helicobacter hepaticus* ATCC51449 (NC004917)*, Campylobacter jejuni* (NC002163)*, Bacillus subtilis* strain 168 (NC000964)*, Treponema pallidum* (NC000919)*, Borrelia burgdorferi* (NC001318)*, Rhodopirellula baltica* SH1 (NC005027) were analysed with the COILS algorithm (Lupas, 1997) as described in Ausmees *et al*. (2003). The coiled coil domain architecture was then determined individually for all proteins containing more than 80 amino acid residues in coiled coil conformation using different settings of the COILS prediction. Proteins exhibiting a potential rod-domain architecture were further analysed with various publicly available bioinformatic tools to detect conserved domains and putative functions.

### *In vitro* filament formation and electron microscopy

N-terminally polyhistidine-tagged proteins (FilP, JNB03975 from *Janibacter* sp. and Mb 1709 from *M. bovis*) were expressed from the pET-28a(+) vector in *E. coli* BL21(DE3) and purified using the Ni-NTA His-Bind® Resin (Novagen) under denaturing conditions (8 M urea) as described in the user manual. To induce filament formation, protein samples were dialysed against a buffer containing 10 mM Tris-HCl with 150 mM NaCl at pH 7.0. Dialysis was performed overnight at 4°C or for 2 h at room temperature with two bath changes. The dialysed samples were then prepared for scanning (SEM) or transmission (TEM) electron microscopy. For SEM the samples were prefixed with 2.5% glutaraldehyde, washed three times in phosphate-buffered saline and fixed in 1% osmium tetraoxide. After dehydration in ethanol the samples were injected through nucleopore filters with 0.2 μm pore size, critical-point dried, mounted on Cambridge alloy Stubbs, silver sputtered and examined in a Zeiss Supra 35-VP field emission SEM equipped with a STEM detector, EDAX Genesis 4000 EDS. For TEM, the dialysed protein samples were applied on carbon-coated and glow discharged grids, negatively stained with 1% uranyl acetate and observed in Hitachi H7100 transmission electron microscope.

### Light microscopy

Samples for phase contrast and fluorescence microscopy were obtained by growing the strains in liquid medium or in the angle between an inserted coverslip and the agar surface (Kieser *et al*., 2000). EGFP fluorescence was observed directly after mounting the coverslips or a drop of liquid culture to a glass slide covered with 1% agarose in phosphate-buffered saline. All fluorescence and phase-contrast microscopy was performed using an Axioplan II imaging fluorescence microscope equipped with appropriate filter sets, an Axiocam charge-coupled device camera and Axiovision software (Carl Zeiss Light Microscopy). Digital images were processed using Axiovision and Adobe Photoshop CS version 8.0 software.

### Atomic force microscopy

Atomic force microscopy of *S. coelicolor* cells was carried out with the MFP3D instrument (Asylum Research) in contact mode in fluid. Cells were hydrated in water during all imaging and force volume measurements. The water reached a maximum temperature of 30°C as monitored with an IR-thermometer. All imaging and force volume measurements were performed using gold coated silicon nitride probes (MLCT-AU) with measured spring constant of 0.05 N m^-1^. A variety of surfaces were tested for culturing cells, including glass, polylysin treated glass, Au-Pd-coated glass and MPS coated glass. The best cell-to-surface adherence was obtained using freshly cleaved muscovite mica (SPI supplies) glued to the glass coverslips. Mica is the surface used as support for all the AFM measurements reported in this paper.
