# Supplementary material for: Intermediate filament-like proteins in bacteria and a cytoskeletal function in Streptomyces
Source: Mol Microbiol. 2008 Oct 9;70(4):1037–50. doi: 10.1111/j.1365-2958.2008.06473.x (PMC2680258; doi:10.1111/j.1365-2958.2008.06473.x)
Supplement: Supplementary file 1 [file mmi0070-1037-SD1.pdf]

Supporting information

Intermediate filament-like proteins in bacteria and a cytoskeletal function in *Streptomyces*

Sonchita Bagchi, Henrik Tomenius, Lyubov Belova and Nora Ausmees

Supporting figures

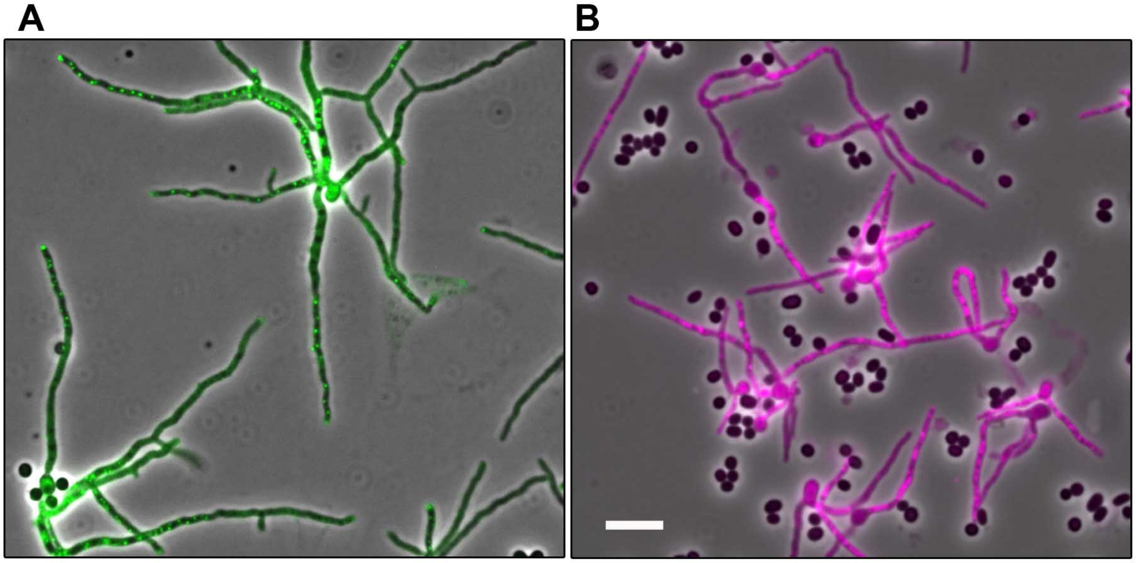

Fig. S1. Localization of putative rod domain proteins SCO5397 and SCO3114 in vegetative hyphae of *S. coelicolor*.
A and B. Overlays of fluorescence and phase contrast micrographs of young vegetative hyphae of the *SCO5397-egfp* (A) and the *SCO3114*$^+$/*SCO3114-mcherry* (B) strains grown for 12 hours in solid MS agar are shown. SCO5397-EGFP fluorescence is is shown in green and SCO3114-mCherry fluorescence is shown in red.

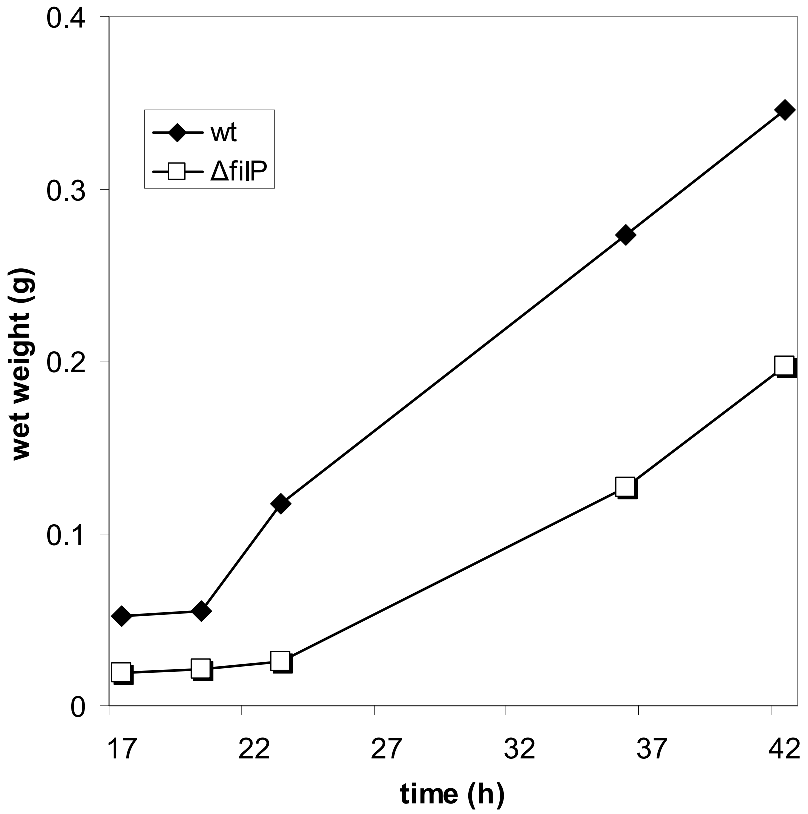

Fig. S2. Growth curves indicate that deletion of *filP* causes delayed germination and reduction of the growth rate. A single representative experiment is shown. Equal amounts of viable spores ($2x10^4$ ml^{-1}) of the wildype and the Δ*filP* strains were inoculated and grown in liquid TSB medium. At indicated times 5 ml aliquots were removed and the wet weight of the bacterial pellet measured (plotted on y axis).

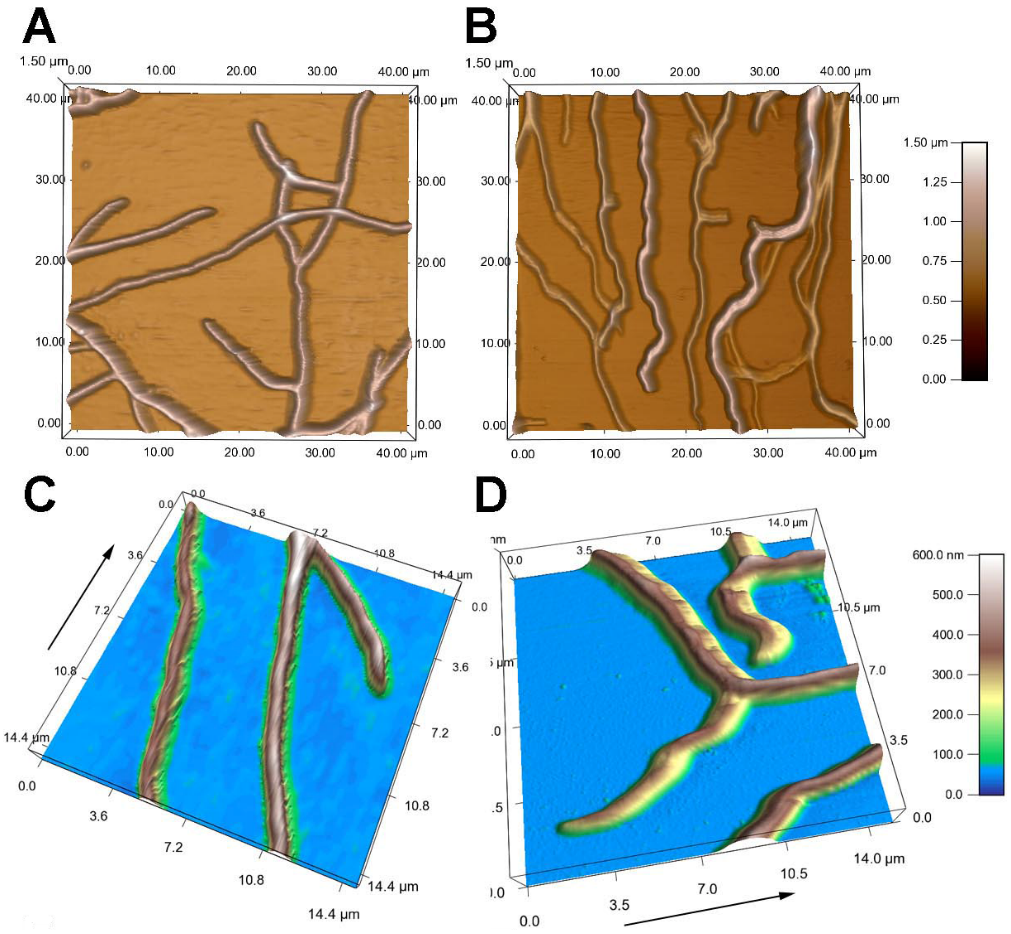

Fig. S3. AFM imaging reveals morphological differences between wildtype and Δ*filP* hyphae.
A-D. 3D-rendered topological images of live hyphae of the wildtype (A, C) and the Δ*filP* strain (B, D). Images were taken using scan size 40x40 µm (A, B) or 14x14 µm (C, D). Different color schemes are used for A-B versus C-D to visualize the height. Black arrows in C and D indicate fast scan direction. E.

Supporting text

Basics of AFM imaging

Imaging is carried out by scanning the tip of the cantilever across the surface of the specimen. Topography is determined by detecting where the tip is in its Z trajectory as the X-Y position of the tip is moved along the surface. The scanning axes are divided into the fast scan axis and slow scan axis. In the display of the data the fast scan direction is displayed as horizontal and slow scan direction as vertical. The fast scan direction is the motion of the scanner that is performed at the frequency of the scan rate. The tip is moved by the Scan size (the size of the image) to collect trace data, and then back to collect retrace data then the scanner advances in the slow scan direction to access each successive scan line (see figure below). This is repeated as many times as required to collect the number of Scan lines that has been requested.

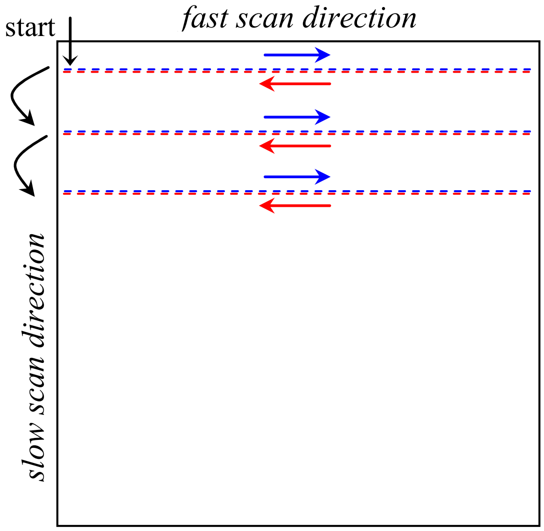

Schematics of the scanning process. Blue and red arrows indicate trace and retrace directions respectively. The distance between the successive lines is exaggerated for clarity.

Supporting table

Table S1. Strains and plasmids

**Strain/plasmid**	**Description**	**Source**
Strain		
S. coelicolor		
M145	Plasmid-free prototroph	(Kieser *et al.*, 2000)
NA335	M145 Δ*filP::[aac(3)IV oriT]*	This study*
NA360	M145 Δ*SCO5397::SCO5397-egfp*, contains only *SCO5397-egfp*	This study
NA399	M145 *SCO3114::SCO3114-mcherry*, merodiploid strain, contains both *SCO3114* and *SCO3114-mcherry*	This study
NA446	M145 Δ*filP::*pNA432 (*filP-egfp*), contains only *filP-egfp*	This study
NA282	M145 *filP::*pNA859[Φ(*filP-egfp*)Hyb], merodiploid strain, contains both *filP* and *filP-egfp*	This study
E. coli		
DH5a	Cloning strain	
DY380	Δ(*mrr-hsdRMS-mcrBC*) *mcrA recA1 λ cI857* Δ(*cro-bio*)<>*tet*, for PCR-targeted mutagenesis	(Lee *et al.*, 2001)
ET12567/pUZ8002	*dam-13::*Tn*9 dcm-6 hsdM*, carries RK2 derivative with defective *oriT* for plasmid mobilization	(Kieser *et al.*, 2000)
GM2929	*dam-13::Tn9 dcm-6 hsdR2 recF143 galK2 galT22 ara-14 lacY1 xyl-5 thi-1 tonA31 rpsL136 hisG4 tsx-78 mtl-1 glnV44 leuB6 rfbD*	M. Marinus
TOP10	Cloning strain	Invitrogen
Plasmid		
pIJ82	Hygromycin-resistant derivative of pSET152	Helen Kieser, JIC, Norwich, UK
pNA559	pET28a with *filP* for expression of His-tagged recombinant protein	This study
pNA663	pET28a with *Janibacter sp*. gene JNB03975 for expression of His-tagged recombinant protein	This study
pNA858	pET28a with *Mycobacterium bovis* gene Mb1709 for expression of His-tagged recombinant protein	This study
pNA859	pEGFP-N2 encoding full-length FilP fused to EGFP	This study
pNA432	Full-length *filP-egfp* in pIJ82	This study
pNA613	Full-length *filP* in pIJ82	This study

*The apramycin resistance cassette was obtained from plasmid pIJ773 and used to first replace *filP* on the cosmid 8F4 (Redenbach *et al.*, 1996) in the *E. coli* strain DY380, which contains an inducible λ RED system (Yu *et al.*, 2000). Mutated cosmid was then introduced into *S. coelicolor* wildtype strain by conjugation (Kieser *et al.*, 2000) and screened for clones where a double recombination event had replaced *filP* by the resistance gene.

Supporting references

Kieser, T., Bibb, M.J., Buttner, M.J., Chater, K.F., and Hopwood, D. (2000) *Practical Streptomyces Genetics.* Norwich: The John Innes Foundation.

Lee, E.C., Yu, D., Martinez de Velasco, J., Tessarollo, L., Swing, D.A., Court, D.L., Jenkins, N.A., and Copeland, N.G. (2001) A highly efficient *Escherichia coli*-based chromosome engineering system adapted for recombinogenic targeting and subcloning of BAC DNA. *Genomics* **73**: 56-65.

Redenbach, M., Kieser, H.M., Denapaite, D., Eichner, A., Cullum, J., Kinashi, H., and Hopwood, D.A. (1996) A set of ordered cosmids and a detailed genetic and physical map for the 8 Mb *Streptomyces coelicolor* A3(2) chromosome. *Mol Microbiol* **21**: 77-96.

Yu, D., Ellis, H.M., Lee, E.C., Jenkins, N.A., Copeland, N.G., and Court, D.L. (2000) An efficient recombination system for chromosome engineering in *Escherichia coli*. *Proc Natl Acad Sci U S A* **97**: 5978-5983.
